# Probiotic Combination of *Bifidobacterium adolescentis* and *Bifidobacterium bifidum* Enhances Lipophagy in MAFLD Rats

**DOI:** 10.1002/mbo3.70100

**Published:** 2025-10-28

**Authors:** Mahtab Mehboodi, Mohammad Mehdi Alinaghi, Maryam Behmanesh, Mohammad Hosein Kolahkaj, Milad Pour Mohammad Ali Namdari, Hadis Khanbabaie, Arezoo Harsjian, Narges Namavar, Saman Rabiei, Maryam Sadat Pishva, Payam Baziyar, Sara Rashki Ghaleno, Mohammad Hasan Maleki

**Affiliations:** ^1^ Department of Medical Microbiology (Bacteriology & Virology), Afzalipour Faculty of Medicine Kerman University of Medical Sciences Kerman Iran; ^2^ Student Research Committee Yasuj University of Medical Sciences Yasuj Iran; ^3^ Department of Food Science and Technology, Faculty of Pharmacy, Tehran Medical Sciences Islamic Azad University Tehran Iran; ^4^ Department of Biology, Central Tehran Branch Islamic Azad University Tehran Iran; ^5^ Neuroscience Research Center Tabriz University of Medical Sciences Tabriz East Azerbaijan Iran; ^6^ Microbiology Department, Faculty of Science Islamic Azad University Arak Branch Tehran Iran; ^7^ Department of Public Health, School of Public Health and safety Shahid Beheshti University of Medical Sciences Tehran Iran; ^8^ Department of Biology Shandiz Institute of Higher Education Mashhad Iran; ^9^ Clinical Research Development Unit, Valiasr Hospital Fasa University of Medical Sciences Fasa Iran; ^10^ Kish International Campus, School of Biology University of Tehran Kish Island Iran; ^11^ Department of Molecular and Cell Biology, Faculty of Basic Science University of Mazandaran Babolsar Iran; ^12^ Department of Cardiology, School of Medicine, Amir al Momenin Hospital Zabol University of Medical Sciences Zabol Iran; ^13^ Department of Clinical Biochemistry, School of Medicine Shiraz University of Medical Sciences Shiraz Iran

**Keywords:** *Bifidobacterium adolescentis*, *Bifidobacterium bifidum*, diabetes, lipophagy

## Abstract

The rise in obesity and type 2 diabetes poses a major public health challenge due to its negative impact on metabolic health and increased risk of complications. Lipophagy, which targets lipid breakdown, is crucial for maintaining lipid balance and may offer therapeutic options for metabolic disorders. Recent studies suggest that probiotics like *Bifidobacterium adolescentis* and *Bifidobacterium bifidum* can positively influence lipid metabolism by affecting lipophagy. This study involved 30 male Sprague Dawley rats divided into five groups: control group, high‐fat diet (HFD) plus streptozotocin (STZ) group, and three groups receiving *B. adolescentis*, *B. bifidum*, or a combination of both probiotics following HFD and STZ induction. Over a 16‐week period, the effects of these probiotics on hepatic lipophagy and lipid profiles were evaluated using stereological and molecular analyses. The probiotic‐treated groups exhibited significant improvements in triglyceride, total cholesterol, and low‐density lipoprotein levels. This was accompanied by an upregulation of autophagy markers (ATG7, LAMP2) and a downregulation of lipogenic markers (PLIN2, FAS, DGAT2, SREBP1). The probiotics effectively modulated lipid accumulation and prevented weight gain. Notably, stereological analysis indicated preserved liver architecture and a reduction in pathological alterations in the treated groups. The results suggest that *B. adolescentis* and *B. bifidum* might enhance hepatic lipophagy and consequently reduce lipid accumulation. This combination can improve metabolic markers, underscoring their therapeutic potential for managing obesity and diabetes. Further research is needed to clarify the specific pathways involved and to assess the clinical applicability of these probiotics in the management of metabolic disorders.

## Graphical abstract

Probiotic supplementation with *Bifidobacterium adolescentis* and *Bifidobacterium bifidum*, especially combined, ameliorates liver injury and metabolic dysfunction in HFD+STZ‐induced MAFLD rats by enhancing lipophagy, preserving liver structure, and improving serum lipid and liver function markers.

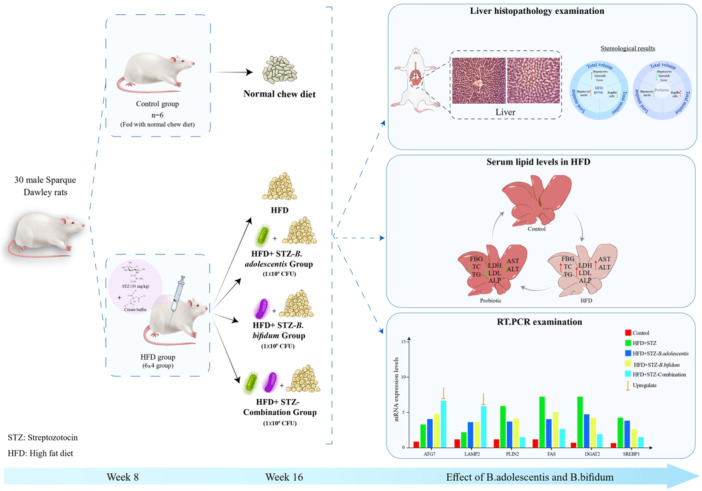

**Abbreviations**
ATG7: autophagy‐related protein 7
DGAT2: diacylglycerol *O*‐acyltransferase 2
FAS: fatty acid synthase
GAPDH: glyceraldehyde 3‐phosphate dehydrogenase
HFD: high‐fat diet
LAMP2: lysosomal‐associated membrane protein 2
LC3: microtubule‐associated protein 1 light chain 3
MAFLD: metabolic‐associated fatty liver disease
NAFLD: nonalcoholic fatty liver disease
NASH: nonalcoholic steatohepatitis
PLIN2: perilipin 2
p62: ubiquitin‐binding protein
SREBP1: sterol regulatory element‐binding protein 1
STZ: streptozotocin
T2D: type 2 diabetes

## 1 Introduction

Obesity and type 2 diabetes (T2D) are raising prevalent chronic conditions that enforce significant global health and economic burdens. The coincidence of these disorders greatly enhances the risk of cardiovascular disease, renal impairment, and hepatic complications. One of the most common liver indications in this context is metabolic‐associated fatty liver disease (MAFLD), a condition characterized by excessive hepatic fat accumulation in the presence of metabolic dysfunction (Sangro et al. 2023; Eslam et al. 2020; Song et al. 2024). MAFLD is strongly associated with insulin resistance, oxidative stress, and inflammation, and its progression can lead to nonalcoholic steatohepatitis (NASH), fibrosis, cirrhosis, and hepatocellular carcinoma (Sergi 2024).

Lipophagy, a specialized form of autophagy targeting lipid droplets (LDs) for lysosomal degradation, plays an essential role in regulating hepatic lipid homeostasis. Through lipophagy, triglycerides (TG) and cholesterol esters stored in LDs are broken down into free fatty acids, which could be utilized for energy production or further metabolic processes (Hwang et al. 2025). Autophagy‐related protein 7 (ATG7) is crucial for autophagosome formation, while lysosomal‐associated membrane protein 2 (LAMP2) mediates autophagosome–lysosome fusion. Impairments in these proteins reduce autophagic flux and promote lipid accumulation (Ren et al. 2024; Han et al. 2023; Yu et al. 2025). Conversely, excessive lipogenesis, driven by transcription factors such as sterol regulatory element‐binding protein 1 (SREBP1) and enzymes including fatty acid synthase (FAS) (Lan et al. 2024; Maleki, Abdizadeh Javazm, et al. 2024) and diacylglycerol *O*‐acyltransferase 2 (DGAT2), contributes to the development and progression of MAFLD (Liu et al. 2022; Longo et al. 2024). Perilipin 2 (PLIN2), a structural protein coating LDs, also influences their stability and degradation, and its overexpression is associated with impaired lipid mobilization (Scorletti et al. 2024).

The gut–liver axis is increasingly recognized as a central regulator of metabolic health. Alterations in the gut microbiota can impair intestinal barrier integrity, facilitate endotoxin translocation, and trigger hepatic inflammation and lipid deposition (Hsu and Schnabl 2023). *Bifidobacterium adolescentis* and *Bifidobacterium bifidum* are probiotic species with demonstrated potential to modulate gut microbiota composition, enhance production of beneficial short‐chain fatty acids, reduce systemic inflammation, and improve lipid metabolism (Meybodi et al. 2025; Segui‐Perez et al. 2025; Long et al. 2021; Lu et al. 2024). Previous studies have reported their beneficial effects on obesity, insulin resistance, and experimental fatty liver; however, the molecular pathways linking these probiotics to hepatic lipophagy remain largely unexplored.

The present study aimed to investigate the effects of individual and combined supplementation with *B. adolescentis* and *B. bifidum* on hepatic lipophagy, lipogenic gene expression, and lipid profiles in a rat model of MAFLD induced by a high‐fat diet (HFD) and streptozotocin (STZ) injection. Additionally, stereological analyses were conducted to assess structural liver alterations beyond standard histological observations.

## 2 Materials and Methods

### 2.1 Cultivation Protocol for *B. adolescentis* and *B. bifidum*

*B. adolescentis* and *B. bifidum* were cultivated in an anaerobic environment using De Man–Rogosa–Sharp (MRS) broth, which was supplemented with 0.25% and 0.05% l‐cysteine hydrochloride, respectively. The cultures were incubated at 37°C, and then 2% (v/v) of each strain was inoculated into the MRS broth.

In addition, agar plates were incubated anaerobically for colony formation, while aerobic plates were maintained to check for contamination. Growth of the cultures was evident within 48 h.

After incubation, the samples were centrifuged at 2500 rpm for 10 min. They were washed three times and then suspended at the appropriate cell concentration. A suspension of *B. adolescentis* and *B. bifidum* was prepared in PBS at a concentration of 1 × 10^9^ CFU in a total volume of 1 mL.

### 2.2 Animals and Experimental Design

Thirty male Sprague Dawley rats, 8 weeks old and weighing between 180 and 200 g, were obtained from the Animal Breeding Center at Shiraz University of Medical Sciences in Shiraz, Iran. The study received approval from the Institutional Animal Ethics Committee of Shiraz University of Medical Sciences (IR.SUMS.AEC.1401.046).

The rats were divided into five groups kept under controlled environmental conditions, including a temperature of 25°C ± 2°C, a relative humidity of 25%–35%, and a cycle of 12 h of light and 12 h of darkness. To allow for acclimatization, the rats were given a standard diet and had unrestricted access to water for 1 week before the experiments commenced.

*Control Group:* Rats receiving a normal diet, administered phosphate‐buffered saline (PBS) via gavage.

*HFD* + *STZ Group:* Rats subjected to an HFD for 16 weeks. A single injection of STZ (Sigma‐Aldrich, USA; Cat. No. S0130) was dissolved in citrate buffer and administered via intraperitoneal injection at 35 mg/kg after 8 weeks of dietary manipulation to induce obesity and T2D.

*HFD* + *STZ‐B. adolescentis Group:* Rats receiving an HFD for 16 weeks, with an STZ injection after 8 weeks of dietary manipulation. After 8 weeks of dietary manipulation, from week 9 to week 16, the rats were given 1 mL of *B. adolescentis* at a concentration of 1 × 10^9^ CFU.

*HFD* + *STZ‐B. bifidum Group:* Rats receiving an HFD for 16 weeks, also receiving an STZ injection after 8 weeks of dietary adjustments. Following the same schedule as the previous group, they were treated with 1 mL of *B. bifidum* at a concentration of 1 × 10^9^ CFU after 8 weeks of dietary manipulation, continuing to week 16.

*HFD* + *STZ‐Combination Group:* Rats receiving an HFD for 16 weeks, with an STZ injection after 8 weeks of dietary manipulation. These rats received a combination of 1 mL of *B. adolescentis* and *B. bifidum*, both at a concentration of 1 × 10^9^ CFU, after 8 weeks of dietary manipulation, continuing to week 16.

**Figure 1:**
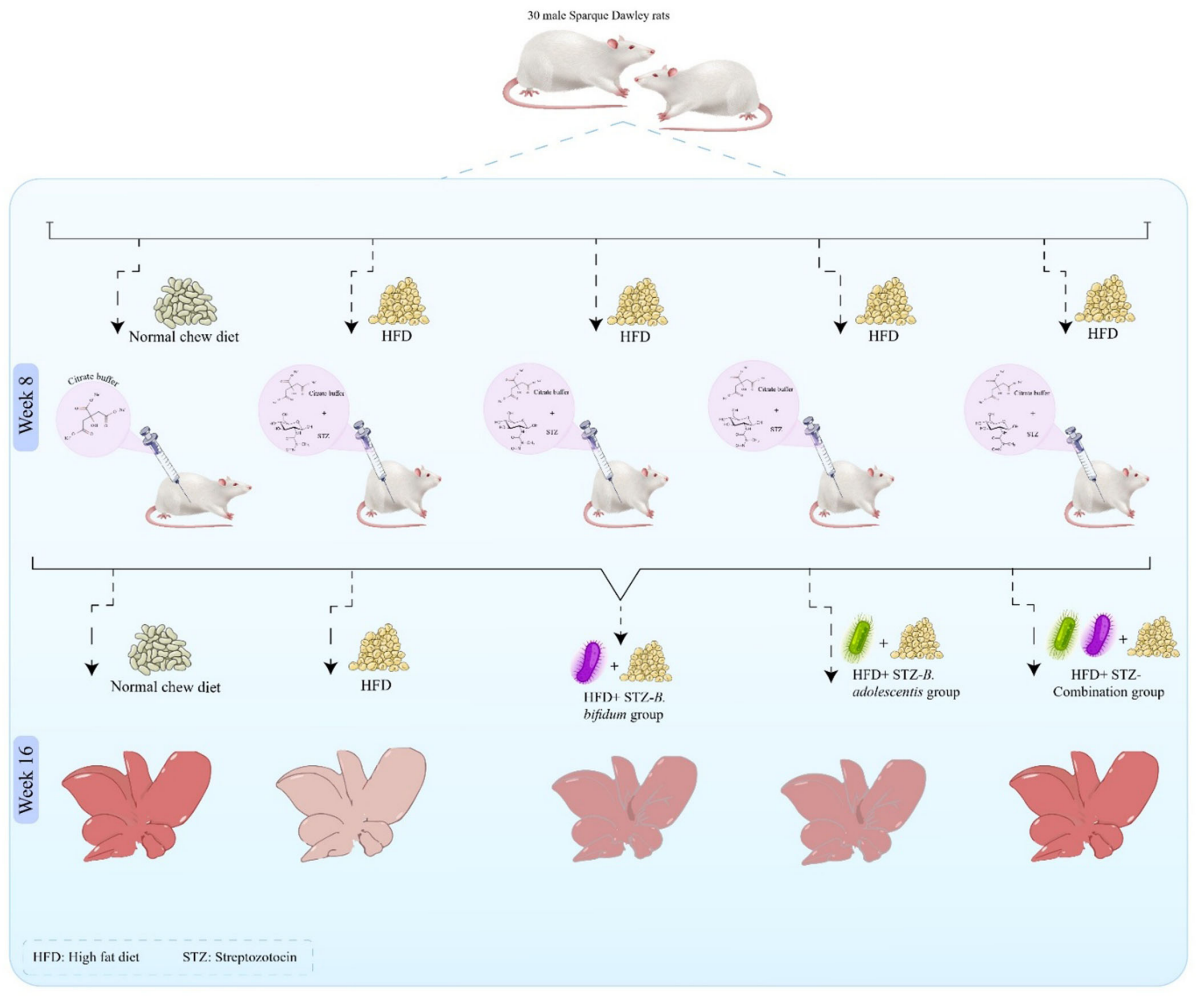
The experimental design of this study. In the control group, the subjects were rats maintained on a standard diet. The high‐fat diet plus streptozotocin (HFD+STZ) group consisted of rats that were fed an HFD for 16 weeks along with STZ. The HFD+STZ‐*Bifidobacterium adolescentis* group included rats that received both the HFD and STZ, supplemented with *B. adolescentis* after 8 weeks of dietary intervention, followed by an additional 8 weeks of treatment with *B. adolescentis* while continuing the HFD. The HFD+STZ‐*Bifidobacterium bifidum* group included rats that received both the HFD and STZ, supplemented with *B. bifidum* after 8 weeks of dietary intervention, followed by an additional 8 weeks of treatment with *B. bifidum* while continuing the HFD. Finally, the HFD+STZ‐combination group included rats that received both the HFD and STZ, supplemented with both *B. bifidum* and *B. adolescentis* after 8 weeks of dietary intervention, followed by an additional 8 weeks of treatment with both *B. bifidum* and *B. adolescentis* while continuing the HFD.

The experimental design of this study is presented in Figure 1.

### 2.3 HFD Preparation

The control diet adhered to the AIN‐93M specifications, comprising 10% of calories from fats, 14% from proteins, and 76% from carbohydrates, with a caloric density of 3.58 kcal/g. In comparison, the HFD derived 50% of its energy from fats, with 10% sourced from soy oil and 40% from sheep's tail fat, while also including 14% from proteins and 36% from carbohydrates, featuring a caloric density of 5.01 kcal/g. The diet's nutritional mixture was based on the described protocol (Reeves et al. 1993; Maleki, Khakshournia, et al. 2024).

### 2.4 Monitoring

The animal body weight was evaluated biweekly throughout the study's duration. Following the final weighing session, the rats were subjected to a fasting period of 6 h before being anesthetized through an intraperitoneal injection of 100 mg/kg of ketamine and 10 mg/kg of xylazine (Merck, Germany). Blood samples were collected from the cardiac region, and the livers were excised and rinsed with cold saline solution. Euthanasia was conducted by placing the rats in a carbon dioxide chamber, adhering to the protocols established by the university's ethics committee.

Upon liver extraction, the liver's weight and volume were assessed to determine the liver index using the formula liver index = (liver weight/body weight × 100), which also enabled additional stereological evaluations. Subsequently, a portion of liver samples was rapidly frozen in liquid nitrogen and stored at −80°C for real‐time polymerase chain reaction (PCR) analyses. Furthermore, a portion of the tissue samples was embedded in a 10% formalin buffer for subsequent stereological examinations.

### 2.5 Biochemical Analyses

Following centrifugation of the blood samples at 3000 rpm for a duration of 15 min, the serum was subsequently frozen at −20°C for future biochemical assays. The evaluation of fasting blood glucose (FBG), TG, total cholesterol (TC), low‐density lipoprotein (LDL), high‐density lipoprotein (HDL), alanine aminotransferase (ALT), aspartate aminotransferase (AST), alkaline phosphatase (ALP), and lactate dehydrogenase (LDH) levels in the serum was conducted using Pars Azmun kits (Pars Azmun Co., Iran) in conjunction with an automated biochemistry analyzer (Prestige 24i, Japan).

### 2.6 Quantitative Reverse Transcriptase‐Quantitative Polymerase Chain Reaction (qPCR) Assessments

The expression levels of messenger RNA (mRNA) for *ATG7*, *LAMP2, PLIN2, FAS, DGAT2*, and *SREBP1 ATG7*, *LAMP2*, *PLIN2*, *FAS*, *DGAT2*, *SREBP1*, *LC3* (*microtubule‐associated protein 1 light chain 3*), and *p62* (ubiquitin‐binding protein) were evaluated using real‐time PCR technique, utilizing the ABI 7500 real‐time PCR system and SYBR Green master mix (Amplicon, Denmark). For this analysis, total RNA was isolated from liver tissue using the TRIzol extraction kit (Yekta Tajhiz Azma, Tehran, Iran) and subsequently reverse‐transcribed into complementary DNA (cDNA) using a cDNA synthesis kit (Yekta Tajhiz Azma, Tehran, Iran) (Siri et al. 2024).

The evaluation of glyceraldehyde 3‐phosphate dehydrogenase was also conducted to act as an endogenous control. Ultimately, the relative quantities of the corresponding mRNA were determined using the comparative *C*_t_ ($2^{-\Delta \Delta C_{t}}$) method. For the primer sequence design, the Allele ID software (version 7.73) was utilized (see Table 1).

**Table 1:** Primer sequences.

Gene	Forward primer	Reverse primer	Accession number
*ATG7*	*GGGGCTTGTACCTCACCAGAT*	*CTCCTCGTCACTCATGTCCCA*	NM_001012097.1
*LAMP2*	*CAGGTGGTTTCCGTGTCTCG*	*CGCTATGGGCACAAGGAAGTT*	NM_017068.3
*PLIN2*	*ATTCTGGACCGTGCCGATTTG*	*TGACATAAGCGGAGGACACCA*	NM_001007144
*FAS*	*ATGGAGGAGGTGGTGATAGCC*	*GACCGCTTAGGCAACCCATAG*	NM_017332.2
*DGAT2*	*ATCAGGTACTCCCGAAGCACA*	*ACTGGAACACGCCCAAGAAAG*	NM_001012345.1
*SREBP1*	*TTAACGTGGGTCTCCTCCGAA*	*TGTCTCACCCCCAGCATAGG*	NM_001276707.1
*LC3*	*GAGTTCTGGTCAGGTTCTCCC*	*GCACCCAAAAGAGCAAGCCTA*	NM_199500.2
*P62*	*ACCCCAGTAAGAGGCTCCAT*	*TCTTGAAGCTCCCCATGTCC*	NM_130405.2
*GAPDH*	*GGAGATTACTGCCCTGGCTCCTA*	*GACTCATCGTACTCCTGCTTGCTG*	NM_017008.4

Abbreviations: ATG7, autophagy‐related protein 7; DGAT2, diacylglycerol *O*‐acyltransferase 2; FAS, fatty acid synthase; GAPDH, glyceraldehyde 3‐phosphate dehydrogenase; LAMP2, lysosomal‐associated membrane protein 2; LC3, microtubule‐associated protein 1 light chain 3; PLIN2, perilipin 2; p62, ubiquitin‐binding protein; SREBP1, sterol regulatory element‐binding protein 1.

### 2.7 Stereological and Histopathological Studies

Unbiased stereology was employed to investigate structural changes in the liver. Along with quantifying these alterations, stereological evaluations were utilized to accurately measure the extent of these changes.

Morphological and stereological analyses were performed using Stereo‐Lite software (SUMS, Shiraz University of Medical Sciences, Iran) integrated with a Nikon E200 light microscope equipped with a high‐resolution digital camera. This software was used for systematic random sampling of histological sections, point counting, and optical dissector measurements, ensuring unbiased and reproducible estimation of liver volumes, hepatocyte numbers, and Kupffer cell (KC) counts.

### 2.8 Estimating Liver Volume

The liver volume (*V*_liver_) was assessed using the Scherle method. This method required suspending the liver with fine threads and immersing it in a container filled with normal saline. The weight of the container was recorded both before and after the addition of the liver. The volume of the liver, represented in cubic centimeters (cm^3^), was determined by subtracting the initial weight from the final weight and then dividing that difference by the specific gravity of normal saline, which is 1.0048 g/cm^3^.

For the stereological and histopathological analyses, the liver tissues were prepared using the orientation method to ensure Isotropic Uniform Random sections. The specimens were fixed in a 10% buffered formaldehyde solution and subsequently dehydrated through a graded series of ethanol, along with a circular sample obtained by punching. The samples of liver were subsequently embedded in paraffin, and sections with thicknesses of 5 and 25 µm were stained with H&E dye (Merck, Germany).

To evaluate the volumetric shrinkage of the tissues, measurements were taken of the circular pieces before and after processing and staining, referred to as “AB” and “AA,” respectively, where “AB” measured 4.5 mm. The formula for calculating the volume shrinkage was subsequently applied:

$$\text{Volume}\;\text{shrinkage}\;=1-{\left(\text{AA}/\text{AB}\right)}^{1.5}.$$

The final volume of the liver was then determined using the subsequent formula (Bordbar et al. 2021):

$$V_{\mathrm{total}\;\mathrm{liver}}=V_{\mathrm{primary}}\times (1-\text{Volume}\;\text{shrinkage}),$$

where *V*_primary_ indicates the initial volume of the liver.

### 2.9 Estimation of Volume Density of Liver Components

**Figure 2:**
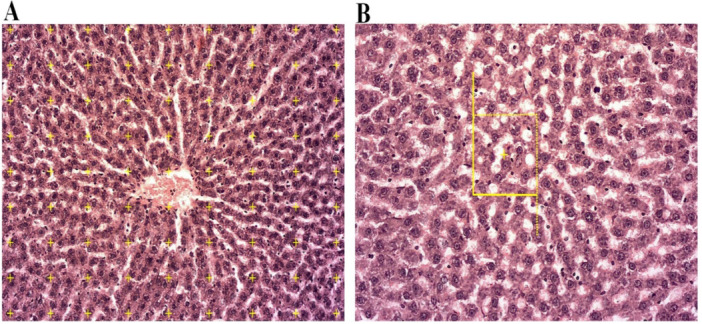
The stereological methods used in this study. (A) A point probe, comprising 81 points, was strategically positioned over the image of the sample section displayed on the monitor. (B) The optical dissector method alongside an unbiased counting frame.

A video‐microscopy system (Nikon E200), manufactured in Japan, was employed to analyze 5‐μm‐thick tissue sections. Subsequently, between 8 and 14 microscopic fields from the liver of each rat were systematically examined and scanned along the *X*‐ and *Y*‐axes at equidistant intervals, utilizing a stage micrometer and achieving a final magnification of ×770. A point probe, comprising 81 points, was then strategically positioned over the image of the sample section displayed on the monitor (refer to Figure 2A). The volume density (*V*_v_) of specific tissue components, including hepatocytes and sinusoids, was determined using the point counting method and the following formula:

$$V_{V(\mathrm{component})}=\Sigma P_{\left(\mathrm{component}\right)}/\Sigma P_{\left(\mathrm{reference}\right)},$$

$$V\;{\text{absolute}}_{\left(\mathrm{component}\right)}=V_{V(\mathrm{component})}\times V_{\left(\mathrm{total}\;\mathrm{liver}\right)}.$$

In this context, Σ*P*_(component)_ and Σ*P*_(reference)_ denote the total points counted within the component's profile and the reference space, respectively.

### 2.10 Estimation of the Number of Hepatocyte Nuclei and KCs

The estimation of hepatocyte nuclei and KCs in tissue sections of 25 μm thickness was conducted using the optical dissector method alongside an unbiased counting frame, under a final magnification of ×1440 (illustrated in Figure 2B). This analysis was performed using a microscope (E200, Nikon) featuring a numerical aperture of 1.30 and a ×40 oil immersion objective, coupled with a microcator (Heidenhain, Germany) and Stereo‐Lite software (SUMS, Iran). The numerical density (*N*_v_) as well as the total counts of hepatocyte nuclei and KCs were subsequently calculated using the following equations:

$$N_{V}=\Sigma Q/(\Sigma P\times \Sigma A\times h)\times \left(t/\mathrm{BA}\right),$$

$$N_{\left(\text{total}\right)}=N_{V}\times V_{\left(\text{final}\;\text{liver}\right)}.$$

Here, ∑*Q* stands for the count of cells measured, ∑*A* refers to the overall area of the unbiased counting frames (with each frame covering an area of 1205.5 µm^2^), *h* indicates the height of the dissector (15 µm), *t* denotes the average thickness of the sections across all examined fields (approximately 22 μm), and BA (Block Advance) signifies the set thickness of the sections processed by the microtome (25 µm).

### 2.11 Statistical Analyses

This study presents mean values along with their standard deviations. Statistical analysis was conducted using version 24.0 of SPSS software, while graphical representations were produced using GraphPad Prism software version 9.5.0. To assess statistical significance among multiple treatment groups, a one‐way analysis of variance (ANOVA) was utilized, followed by the Tukey post hoc test. Additionally, a two‐way repeated measures ANOVA was applied to evaluate changes in the body weight of experimental rats over time, accounting for the interaction between treatment and time. A *p* < 0.05 was considered statistically significant.

The sample size for each experimental group (*n* = 6) was determined using a priori power analysis in G*Power software (Universität Düsseldorf, Germany). Calculations were based on an effect size (*f*) of 0.55 estimated from preliminary pilot data and previous studies investigating similar biochemical and molecular outcomes in HFD and STZ‐induced rodent models. We assumed a statistical power (1 − *β*) of 80% and a significance level (*α*) of 0.05 for detecting group differences using one‐way ANOVA. The analysis indicated that a minimum of six animals per group was required to reliably detect biologically relevant changes while minimizing animal use in accordance with the 3Rs principle.

## 3 Results

### 3.1 Combined Treatment of *B. adolescentis* and *B. bifidum* Modulated the Increase and STZ‐Induced Decrease of Body Weight

**Figure 3:**
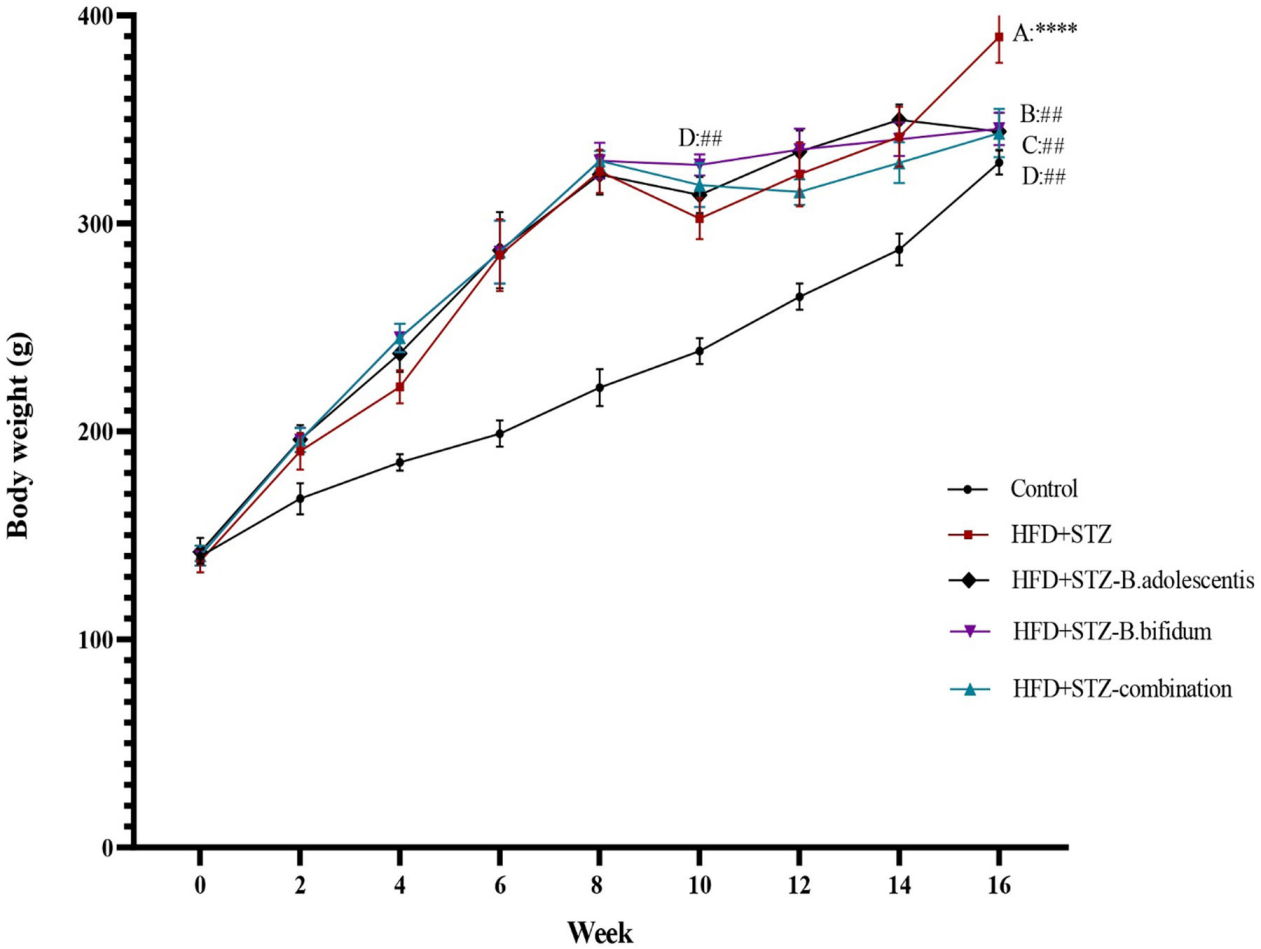
The variations in body weight of rats over the course of a 16‐week experimental period. *The control group versus the high‐fat diet plus streptozotocin (HFD+STZ) rats, and ^#^HFD+STZ group versus the other experimental groups. The symbols * and ^#^ represent *p* < 0.05, while ** and ^##^ signify *p* < 0.01. Similarly, *** and ^###^ indicate *p* < 0.001. In the control group, the subjects were rats maintained on a standard diet. The HFD (HFD+STZ) group consisted of rats that were fed an HFD for 16 weeks along with STZ. The HFD+STZ‐*Bifidobacterium adolescentis* group included rats that received both the HFD and STZ, supplemented with *B. adolescentis* after 8 weeks of dietary intervention, followed by an additional 8 weeks of treatment with *B. adolescentis*, while continuing the HFD. The HFD+STZ‐*Bifidobacterium bifidum* group included rats that received both the HFD and STZ, supplemented with *B. bifidum* after 8 weeks of dietary intervention, followed by an additional 8 weeks of treatment with *B. bifidum* while continuing the HFD. Finally, the HFD+STZ‐combination group included rats that received both the HFD and STZ, supplemented with both *B. bifidum* and *B. adolescentis* after 8 weeks of dietary intervention, followed by an additional 8 weeks of treatment with both *B. bifidum* and *B. adolescentis* while continuing the HFD. All data are presented as means ± standard deviation (SD) with a sample size of *n* = 6.

As shown in Figure 3, after 8 weeks, the weight of rats that were given an HFD rose considerably when compared with the control group. However, following the injection of STZ, a severe weight loss was observed in the groups receiving STZ (HDF+STZ), with a more pronounced reduction in those that did not receive probiotics. Rats that were fed a combination of two probiotic strains exhibited less weight loss compared with the HFD group (*p* < 0.01). Ultimately, all probiotic‐receiving groups had lower weights than those on the HFD and the STZ‐induced diabetic groups (*p* < 0.01).

### 3.2 Combined Treatment of *B. adolescentis* and *B. bifidum* Improved Lipid and Liver Marker

As illustrated in Table 2, serum levels of FBG exhibited a significant increase in the HFD (HFD+STZ) group when compared with the control (*p* < 0.001). While the individual and combined treatments of *B. adolescentis* and *B. bifidum* led to reductions in serum FBG levels within the HFD+STZ‐*B. adolescentis*, HFD+STZ‐*B. bifidum*, and HFD+STZ‐combination groups compared with the HFD+STZ control group, these observed alterations did not reach statistical significance.

**Table 2:** Evaluation of lipid profiles and liver biochemical markers across different groups of rats.

Biochemical parameters	Control	HFD	HFD‐*Bifidobacterium adolescentis*	HFD‐*Bifidobacterium bifidum*	HFD‐combination
FBG (mg/dL)	90.15 ± 4.2	248.3 ± 14.2***	233.6 ± 12.06	225.4 ± 9.25	203.3 ± 11
TG (mg/dL)	48.24 ± 5.79	82.21 ± 7.23***	65.42 ± 6.4^#^	60.26 ± 5.9^#^	54.19 ± 4.8^##^
TC (mg/dL)	62.23 ± 4.15	125 ± 6.11***	90.13 ± 6.2^#^	86.26 ± 5.12^##^	73.41 ± 6.2^T^
HDL (mg/dL)	43.5 ± 4.25	31.33 ± 3.19*	33.44 ± 3.11	35.22 ± 4.1	39.23 ± 3.5^#^
LDL (mg/dL)	23.5 ± 4.1	68.94 ± 4.21***	52 ± 5.2^#^	56.33 ± 5.8^#^	42.19 ± 4.8^##,$^
ALT (IU/L)	38 ± 4.3	62 ± 4.24***	51.32 ± 4.83^#^	45.46 ± 5.8^##^	43.3 ± 4.6^##^
AST (IU/L)	43.26 ± 4.2	104 ± 7.65***	72 ± 8.3^#^	77.5 ± 9.2^#^	53.4 ± 6.1^$,T,^^##^
ALP (IU/L)	463.7 ± 43.9	846.5 ± 77.8***	701.2 ± 40.9^#^	671 ± 44.8^#^	532 ± 41.4^$$,T,^^##^
LDH (IU/L)	979.3 ± 55	1377 ± 82***	1165 ± 68	1205 ± 70.4	1050 ± 69.4^$$,T,#^

*Note:* Data are presented as mean ± standard deviation (*n* = 6). *Control versus high‐fat diet plus streptozotocin (HFD+STZ) group. ^#^HFD+STZ versus other experimental groups. ^$,T^HFD+STZ‐combination versus HFD+STZ‐*B. bifidum* and HFD+STZ‐*B. adolescentis*, respectively. Symbols ^*,#,$,T^denote *p* < 0.05; ^##,$$,TT^denote *p* < 0.01; ^***,###,$$$,TTT^ denote *p* < 0.001.

Control group: Rats on a standard diet. HFD+STZ group: Rats fed an HFD for 16 weeks + STZ. HFD+STZ‐*B. adolescentis* group: The same diet+STZ, then 8 weeks with *B. adolescentis*. HFD+STZ‐*B. bifidum* group: The same diet+STZ, then 8 weeks with *B. bifidum*. HFD+STZ‐Combination group: The same diet+STZ, then 8 weeks with both *B. bifidum* and *B. adolescentis*.

Abbreviations: ALP, alkaline phosphatase; ALT, alanine aminotransferase; AST, aspartate aminotransferase; FBG, fasting blood glucose; HDL, high‐density lipoprotein; LDH, lactate dehydrogenase; LDL, low‐density lipoprotein; TC, total cholesterol; TG, triglycerides.

Additionally, Table 2 indicates that the serum levels of TG, TC, and LDL were significantly increased in the HFD+STZ group when compared with the control group (*p* < 0.0001). Conversely, both individual and combined administrations of *B. adolescentis* and *B. bifidum* resulted in notable reductions in TG (*p* < 0.05, *p* < 0.05, and *p* < 0.01), TC (*p* < 0.05, *p* < 0.01, and *p* < 0.01), and LDL (*p* < 0.05, *p* < 0.05, and *p* < 0.01) relative to the HFD+STZ group. Furthermore, the combined treatment of *B. adolescentis* and *B. bifidum* significantly decreased TC (*p* < 0.05) and LDL levels (*p* < 0.05) compared with the HFD+STZ‐*B. adolescentis* and HFD+STZ‐*B. bifidum* groups, respectively. The HDL serum level was significantly lower in the HFD+STZ group compared with the control group (*p* < 0.05), while its level in the HFD+STZ‐combination group was significantly higher than that of the HFD+STZ group (*p* < 0.05).

The variations in serum levels of liver indices, including ALT, AST, ALP, and LDH across all groups were evaluated. As outlined in Table 2, the serum levels of ALT, AST, ALP, and LDH were elevated in the HFD+STZ group compared with the control group (*p* < 0.001). However, both individual and combined administrations of *B. adolescentis* and *B. bifidum* resulted in notable reductions in ALT (*p* < 0.05, *p* < 0.01, and *p* < 0.01), AST (*p* < 0.05, *p* < 0.05, and *p* < 0.01), ALP (*p* < 0.05, *p* < 0.05, and *p* < 0.01), and LDH (ns and *p* < 0.05) relative to the HFD+STZ group. Furthermore, the combined treatment of *B. adolescentis* and *B. bifidum* in the HFD+STZ‐combination group significantly decreased AST (*p* < 0.05, *p* < 0.05), ALP (*p* < 0.01, *p* < 0.05), and LDH levels (*p* < 0.05, *p* < 0.01) compared with HFD+STZ‐*B. bifidum* and HFD+STZ‐*B. adolescentis* groups, respectively.

### 3.3 Combined Treatment With *B. bifidum* and *B. adolescentis* Enhanced Lipophagy and Decreased Lipid Biosynthesis Gene Markers in the Liver of HFD Fed and STZ Injected Rats

**Figure 4:**
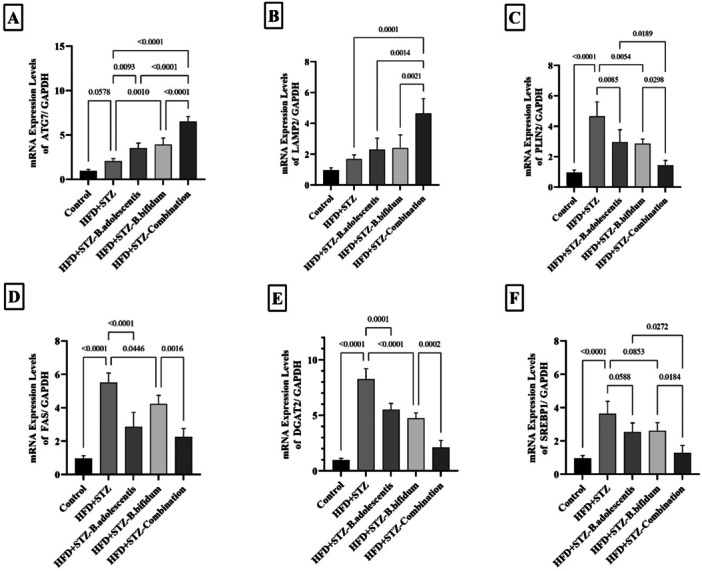
The relative gene expression levels of ATG7, LAMP2, PLIN2, FAS, DGAT2, and SREBP1 genes were sampled from the liver samples of the examined rats. (A) ATG7, (B) LAMP2, (C) PLIN2, (D) FAS, (E) DGAT2, and (F) SREBP1. In the control group, the subjects were rats maintained on a standard diet. The high‐fat diet plus streptozotocin (HFD+STZ) group consisted of rats that were fed an HFD for 16 weeks along with STZ. The HFD+STZ‐*Bifidobacterium adolescentis* group included rats that received both the HFD and STZ, supplemented with *B. adolescentis* after 8 weeks of dietary intervention, followed by an additional 8 weeks of treatment with *B. adolescentis* while continuing the HFD. The HFD+STZ‐*Bifidobacterium bifidum* group included rats that received both the HFD and STZ, supplemented with *B. bifidum* after 8 weeks of dietary intervention, followed by an additional 8 weeks of treatment with *B. bifidum* while continuing the HFD. Finally, the HFD+STZ‐combination group included rats that received both the HFD and STZ, supplemented with both *B. bifidum* and *B. adolescentis* after 8 weeks of dietary intervention, followed by an additional 8 weeks of treatment with both *B. bifidum* and *B. adolescentis* while continuing the HFD. The significance between the compared groups is displayed above the column of each Bar Chart. All data are presented as mean ± SD (*n* = 6). ATG7, autophagy‐related protein 7; DGAT2, diacylglycerol *O*‐acyltransferase 2; FAS, fatty acid synthase; LAMP2, lysosomal‐associated membrane protein 2; PLIN2, perilipin 2; SREBP1, sterol regulatory element‐binding protein 1.

As depicted in Figure 4, there was a notable rise in the mRNA levels of FAS, PLIN2, DGAT2, and SREBP1 in the HFD+STZ group in comparison to the control (*p* < 0.001). Conversely, both the HFD‐*B. adolescentis* and HFD‐*B. bifidum* groups exhibited a significant reduction in the mRNA expression levels of these genes compared with the HFD+STZ group. Individual treatments with *B. adolescentis* and *B. bifidum* were able to reduce the expression of these genes, while the combination treatment specifically reversed the enhanced expression to normal levels when compared with the HFD‐*B. adolescentis* and HFD‐*B. bifidum* groups. The expression of ATG7 and LAMP2 was slightly (nearly significant) upregulated in rats fed an HFD and injected with STZ. Analyses using real‐time PCR indicated that both individual and specifically combined treatments of *B. adolescentis* and *B. bifidum* could upregulate the expression of ATG7 and LAMP2 compared with the HFD+STZ group.

### 3.4 LC3 and p62 mRNA Expression in Hepatic Tissue

**Figure 5:**
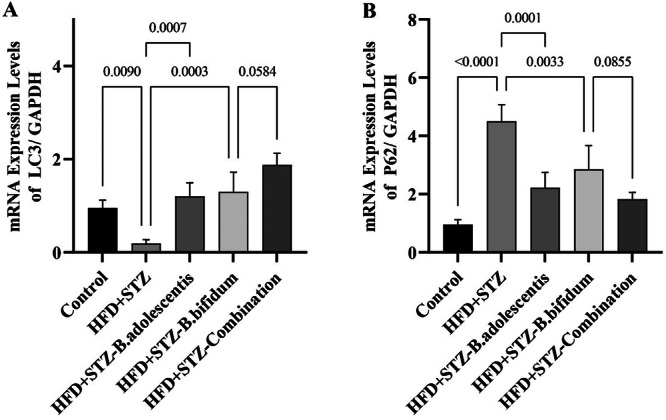
Effects of *Bifidobacterium adolescentis* and *Bifidobacterium bifidum* on hepatic LC3 and p62 messenger RNA (mRNA) expression in high‐fat diet plus streptozotocin (HFD+STZ)‐induced MAFLD rats. (A) Relative mRNA expression levels of LC3 normalized to GAPDH. (B) Relative mRNA expression levels of p62 normalized to GAPDH. Data are presented as mean ± SD (*n* = 6 per group). Statistical significance was determined by one‐way analysis of variance followed by Tukey's post hoc test. *p* values are indicated above the bars; *p* < 0.05 was considered statistically significant. GAPDH, glyceraldehyde 3‐phosphate dehydrogenase; MAFLD, metabolic‐associated fatty liver disease.

To investigate the effect of probiotic supplementation on hepatic autophagy, the mRNA expression levels of LC3 and p62 were measured in liver tissue (Figure 5). The HFD+STZ group exhibited a significant reduction in LC3 expression compared with the control group (*p* = 0.0090) and a significant increase in p62 expression (*p* < 0.0001), indicating suppressed autophagic activity. Supplementation with *B. adolescentis* significantly increased LC3 expression compared with the HFD+STZ group (*p* = 0.0007) and significantly reduced p62 expression (*p* = 0.0001). Similarly, *B. bifidum* administration significantly increased LC3 levels (*p* = 0.0003) and decreased p62 levels (*p* = 0.0033) compared with the HFD+STZ group. The combination of *B. adolescentis* and *B. bifidum* produced a comparable trend, with LC3 expression approaching significance (*p* = 0.0584) and p62 expression showing a borderline reduction (*p* = 0.0855) relative to the HFD+STZ group. These results suggest that both monotherapy and combination therapy with these probiotics may enhance hepatic autophagic flux in MAFLD rats.

### 3.5 Cotreatment of *B. bifidum* and *B. adolescentis* Reversed Liver Weight and Liver Index to Normal

**Figure 6:**
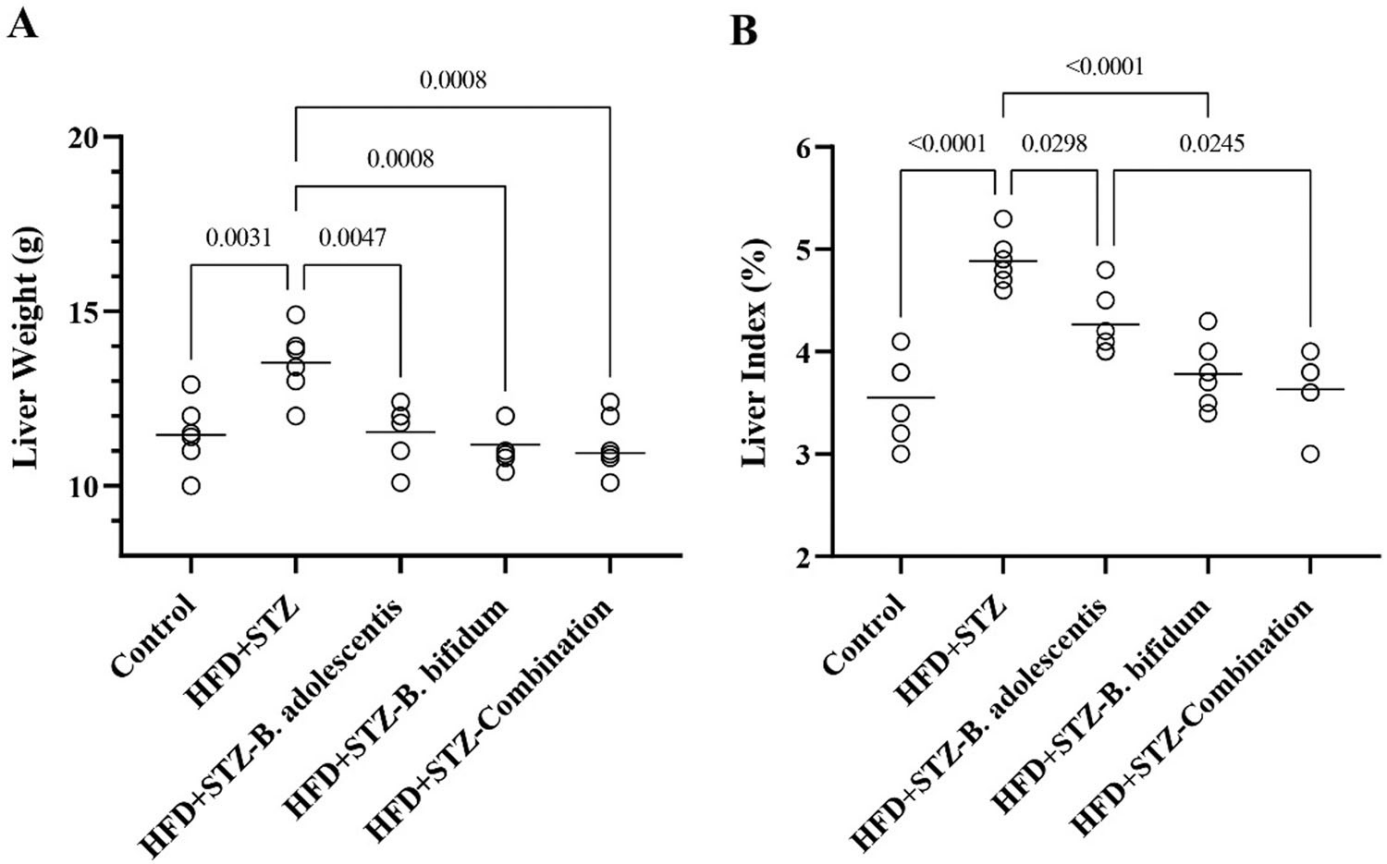
Liver weight and liver index. The study involved measuring liver weight (A) and liver index (B) among rats. Specifically, the liver weight (in grams) and liver index (as a percentage) were recorded, with results expressed as mean ± standard deviation (*n* = 6). The control group consisted of rats fed a standard diet. The high‐fat diet plus streptozotocin (HFD+STZ) group included rats that underwent an HFD for 16 weeks in conjunction with STZ treatment. The HFD+STZ‐*Bifidobacterium adolescentis* group featured rats that received the HFD and STZ, along with supplementation of *B. adolescentis* starting after 8 weeks, continuing for an additional 8 weeks while maintaining the HFD. The HFD+STZ‐*Bifidobacterium bifidum* group had rats on the HFD and STZ supplemented with *B. bifidum* after 8 weeks and continued the treatment for another 8 weeks. Finally, the HFD+STZ‐Combination group consisted of rats that received both the HFD and STZ, supplemented with both *B. bifidum* and *B. adolescentis* after the initial 8 weeks, followed by an additional 8 weeks of treatment with both probiotics while on the HFD.

As shown in Figure 6, the liver weight in the HFD+STZ group was considerably higher than that seen in the control group (*p* < 0.01). Treatment with *B. adolescentis* and *B. bifidum* in the HFD+STZ‐*B. adolescentis*, HFD+STZ‐*B. bifidum*, and HFD+STZ‐combination groups resulted in a notable reduction in liver weights when compared with the HFD+STZ group (*p* < 0.01) (Figure 5A). Regarding the liver index, which is determined by dividing the liver weight of each rat by its total body weight and multiplying by 100 (Figure 5B), the HFD+STZ group exhibited a higher liver index (4.9% ± 0.29%) relative to the control group (3.53% ± 0.24%) (*p* < 0.0001). Conversely, the HFD+STZ‐*B. adolescentis*, HFD+STZ‐*B. bifidum*, and HFD+STZ‐combined groups displayed lower liver indices (4.3% ± 0.23%, 3.9% ± 0.21%, and 3.4% ± 0.19%, respectively) in comparison to the HFD+STZ group (*p* < 0.05, *p* < 0.01, and *p* < 0.001, respectively).

### 3.6 Cotreatment of *B. bifidum* and *B. adolescentis* Could Improve Liver Volume and Stereological Indices

**Figure 7:**
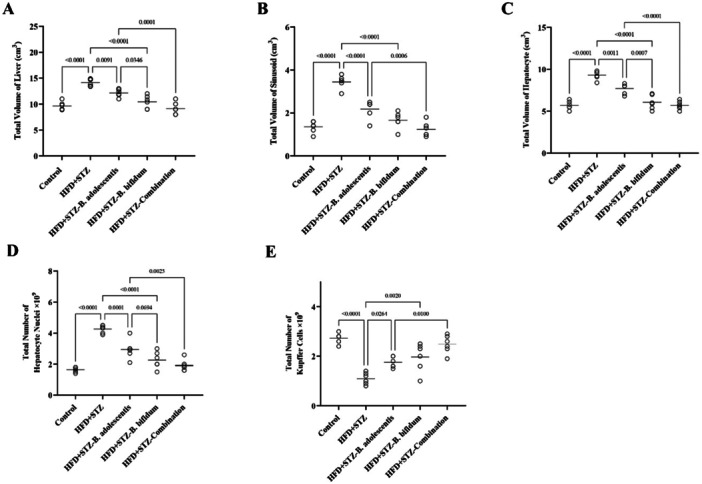
Liver's quantitative metrics. (A) The overall liver volume (cm^3^), while panel (B) represents the total sinusoidal volume (cm^3^). (C) The total volume of hepatocytes (cm^3^), followed by panel (D), which indicates the total count of hepatocyte nuclei. Lastly, panel (E) details the overall number of Kupffer cells present. All data are presented as mean ± standard deviation (*n* = 6). In the control group, the subjects were rats maintained on a standard diet. The high‐fat diet plus streptozotocin (HFD+STZ) group consisted of rats that were fed an HFD for 16 weeks along with STZ. The HFD+STZ‐*Bifidobacterium adolescentis* group included rats that received both the HFD and STZ, supplemented with *B. adolescentis* after 8 weeks of dietary intervention, followed by an additional 8 weeks of treatment with *B. adolescentis* while continuing the HFD. The HFD+STZ‐*Bifidobacterium bifidum* group included rats that received both the HFD and STZ, supplemented with *B. bifidum* after 8 weeks of dietary intervention, followed by an additional 8 weeks of treatment with *B. bifidum* while continuing the HFD. Finally, the HFD+STZ‐combination group included rats that received both the HFD and STZ, supplemented with both *B. bifidum* and *B. adolescentis* after 8 weeks of dietary intervention, followed by an additional 8 weeks of treatment with both *B. bifidum* and *B. adolescentis* while continuing the HFD.

The quantitative liver parameters presented in Figure 7 indicate that the total liver volume in the HFD group was significantly elevated by 25% in comparison to the control group (*p* < 0.001) (Figure 6A). Furthermore, the volumes of sinusoids and hepatocytes in the HFD group exhibited increases of 1.6‐fold and 45%, respectively, in comparison to the control group (*p* < 0.001) (Figure 6B,C). Notably, the administration of *B. adolescentis* resulted in a significant reduction in total liver volume by 20% (*p* < 0.01), as well as an 80% decrease in sinusoidal volume and a 22% decrease in hepatocyte volume (*p* < 0.001, *p* < 0.01) among the HFD+STZ‐*B. adolescentis* rats. Likewise, the administration of *B. bifidum* led to a 33% reduction in total liver volume (*p* < 0.001), an 89% reduction in sinusoid volume (*p* < 0.001), and a 70% reduction in hepatocyte volume (*p* < 0.01) in the HFD+STZ‐*B. bifidum* group compared with the HFD group.

In contrast, the number of hepatocyte nuclei in the HFD group increased by 2.2‐fold relative to the control group (*p* < 0.001), but this number reduced by onefold and 1.65‐fold after treatment with *B. adolescentis* and *B. bifidum* in the HFD+STZ‐*B. adolescentis* (*p* < 0.001) and HFD+STZ‐*B. bifidum* (*p* < 0.001) groups, respectively (Figure 6D). Moreover, the quantity of KCs was significantly reduced by 1.5‐fold in the HFD+STZ group compared with the control group (*p* < 0.001). However, this decrease was notably reversed by 62% and 73% following treatment with *B. adolescentis* and *B. bifidum* in the HFD+STZ‐*B. adolescentis* (*p* < 0.05) and HFD+STZ‐*B. bifidum* (*p* < 0.01) groups, respectively (Figure 6E). Additionally, the combined treatment of both *Bifidobacterium* strains restored all aforementioned parameters to normal levels.

## 4 Discussion

In this study, we examined the impacts of *B. adolescentis* and *B. bifidum* on metabolic parameters in STZ‐induced diabetic rats subjected to an HFD. Our findings demonstrate that the separate and combined administration of these probiotics significantly prevented weight gain and improved blood lipid profiles, likely through the modulation of lipophagy in the liver.

The notable reduction in TG, TC, and LDL concentrations might indicate a cardioprotective effect of these strains, emphasizing their potential as therapeutic agents in managing metabolic disorders. Although we did not observe significant changes in blood glucose levels, the reduced lipid profile suggests that these probiotics may selectively influence certain metabolic pathways, highlighting the complexity of metabolic regulation in the context of obesity and diabetes.

Mechanistically, our data suggest an involvement of specific molecular pathways in mediating the observed benefits. The upregulation of autophagy‐related proteins, particularly *ATG7* and *LAMP2*, in the liver may indicate enhanced lipophagy, a process crucial for the degradation of LDs and the maintenance of cellular lipid homeostasis. Conversely, the downregulation of *PLIN2*, *FAS*, *DGAT2*, and *SREBP1* points to a potential shift in lipid metabolism, wherein de novo lipogenesis and lipid accumulation are curtailed. Such alterations may contribute to the observed improvements in the lipid profile and overall liver function.

Furthermore, the protective effects of these strains against liver damage are underscored by the normalization of serum liver enzyme levels, including AST, ALT, and LDH. Stereological analyses reveal that treatment with these probiotics mitigates the pathological changes associated with diabetes and obesity, including alterations in hepatic architecture and the proliferation of KCs. The restoration of the liver index further corroborates the regenerative potential of *B. adolescentis* and *B. bifidum*.

Recent studies have highlighted the role of gut microbiota in influencing metabolic health, particularly in the development and management of metabolic disorders, such as obesity and T2D. The administration of specific probiotic strains has been associated with improved metabolic parameters, including reductions in body weight and favorable modifications to lipid profiles (Van Syoc et al. 2023). Investigations into *Bifidobacterium* strains reveal their significant impacts on lipid metabolism, particularly in HFD‐induced models of metabolic disorders. The administration of *B. adolescentis* and *B. bifidum* has been shown to prevent weight gain and enhance blood lipid profiles (L. Wang, Jiao, et al. 2021). Notably, reductions in TG, TC, and LDL levels suggest a hepatoprotective role, emphasizing these probiotics as potential therapeutic agents for lipid‐related conditions (Lu et al. 2024; G. Wang et al. 2020).

L. Wang, Jiao, et al. (2021) examined the impact of various bifidobacteria on MAFLD by focusing on hepatocellular injury, liver fat accumulation, liver inflammation, and histopathological changes in the liver. Both *B. adolescentis* and *B. bifidum* influence MAFLD primarily by altering the intestinal microbiota, enhancing the levels of propionic acid and butyric acid, regulating lipid metabolism and gut permeability, ultimately leading to a reduction in liver inflammation and fat accumulation. In a comparative study, Qian et al. (2022) discovered that strains of *B. adolescentis* were more effective in alleviating symptoms of T2D compared with *B. bifidum* strains. This superior effect is closely linked to *B. adolescentis*'s capability to restore balance in the intestinal microbiota, boost the population of short‐chain fatty acid‐producing bacteria, and decrease inflammation in mice with T2D. B. Wang, Kong, et al. (2021) demonstrated that strains of *B. adolescentis* elevated serum leptin levels and stimulated the expression of genes associated with thermogenesis and lipid metabolism in brown adipose tissue. It is suggested that certain strains of *B. adolescentis* may help mitigate obesity and alter the intestinal microbiota in mice. We also noted a reduction in lipid profile levels and improvements in the liver panel, which indicated a decrease in liver injury caused by an HFD and STZ administration. This beneficial effect was observed following the administration of both *B. adolescentis* and *B. bifidum*, as well as their combined use, which proved to be more effective than the use of each probiotic alone.

We hypothesized that the beneficial effects of these probiotics may be attributed to the activation of lipophagy in the liver, a process that has received limited attention in research. Lipophagy, a process involving the selective degradation of LDs within cells via autophagy, plays a crucial role in lipid metabolism, especially in the liver. The importance of lipophagy has garnered attention due to its potential influence on lipid homeostasis and metabolic disorders, such as MAFLD. Lipophagy allows the liver to manage lipid accumulation by breaking down stored fats when energy reserves are low or when confronted with excess lipids. This process is particularly vital during states of high‐fat intake, as it helps prevent the detrimental effects of excessive lipid storage, including inflammation and insulin resistance (Li et al. 2021; Carotti et al. 2020). Minami et al. (2023) demonstrated that stimulating hepatocyte lipophagy in vivo significantly mitigated both steatosis and hepatitis in a mouse model of diet‐induced NASH. The mechanism underlying this effect involves active lipophagy facilitating the clearance of lipids from hepatocytes. This process minimizes the detrimental buildup of intracellular nonesterified fatty acids, which ultimately leads to a decrease in liver fatty acid levels along with a reduction in associated inflammation and fibrosis. Additionally, lipophagy plays a significant role in fat mobilization. It may serve as a connection between T2D and lipid dysregulation by influencing LDs. Furthermore, lipophagy can mimic the effects of nutrient restriction, which has been recognized as one of the most effective methods for reducing visceral fat in both animal studies and human populations (Huang et al. 2021).

Hepatic ATG7 is essential for initiating autophagy and lipophagy, and it plays a crucial role in the regulation of hepatic lipid metabolism (Shin 2020). Zhao et al. (2020) demonstrated that lipophagy impairment in mice can lead to increased endoplasmic reticulum (ER) stress in the liver, which in turn worsens insulin tolerance. Yang et al. (2010) found that the liver‐specific overexpression of Atg7 in obese mice reinstates insulin receptor signaling within liver tissue. This intervention reduces obesity‐related ER stress and enhances both glucose tolerance and insulin sensitivity. In another study, Barrientos‐Riosalido et al. (2023) investigated the expression of hepatic ATG7 mRNA and ATG7 protein in the context of obesity‐related MAFLD. Their findings indicate that ATG7‐mediated autophagy may be crucial in the development of MAFLD, particularly in NASH, potentially serving a protective function. Xiao et al. (2016) revealed that ERK1/2 mitigates liver steatosis in leptin receptor‐deficient (db/db) mice by enhancing ATG7‐dependent autophagy. It has been noted that the overproduction of PLIN2 interferes with the function of ATGL, which is essential for the breakdown of TG into free fatty acids (Chandrasekaran et al. 2024). The regulatory function of PLIN2 was illustrated by the observation that Plin2 knockout mice did not experience hepatic steatosis when exposed to an HFD. This regulatory role is associated with its function in LDs and decreased availability for lipolytic enzymes (Tao and Xu 2020). The breakdown of these proteins enables ATGL to reach LDs and may aid in the process of lipolysis (Sathyanarayan et al. 2017). Furthermore, the removal of PLIN2, the most prevalent protein linked to fat droplets in a fatty liver, offers protection to mice from diet‐induced MAFLD (Griffin et al. 2021). Tsai et al. (2017) demonstrated that Plin2−/− mice exhibit a 60% decrease in TG levels and are safeguarded against fatty liver disease. The overexpression of PLIN2 helps to shield LDs from macroautophagy/autophagy, while a lack of PLIN2 leads to increased autophagy and lower hepatic TG levels. The heightened autophagy observed in Plin2−/− mice provides protection against severe hepatosteatosis and hepatocyte apoptosis triggered by ER stress. In a separate study, Ji et al. (2019) noted an increase in both PLIN2 mRNA and protein levels in the β‐cells of patients with T2D. They also observed significant alterations in the expression of genes related to lipid metabolism, apoptosis, and oxidative stress. The elevated accumulation of LD in β‐cells of T2D patients was linked to the suppression of nuclear translocation of TFEB, which is the primary regulator of autophagy, as well as a decrease in the expression of the lysosomal biomarker LAMP2. LAMP2B engages with phosphatidic acid, enabling interactions between lysosomes and LDs, and enhances lipid breakdown through microlipophagy, contingent upon the endosomal sorting complexes essential for transport (Sakai et al. 2024). Ren and colleagues demonstrated that fatty acids impede autophagy flux mediated by LAMP2 by activating the ER stress pathway in the context of alcohol‐related liver disease (Ren et al. 2024). These findings indicate that the degradation process of autophagosomes and lysosomes mediated by Lamp‐2 plays a role in the development of diabetes related to obesity (Yasuda‐Yamahara et al. 2015). The lysosomal membrane protein LAMP2B is responsible for facilitating microlipophagy, which is aimed at addressing obesity‐related conditions. Furthermore, increased levels of LAMP2B in mice help avert obesity, insulin resistance, and inflammation in adipose tissue that are typically induced by an HFD (Sakai et al. 2024).

In the current experiment, a slight increase in ATG7 and LAMP2 mRNA expression, alongside a marked elevation of PLIN2, was observed in the obese and diabetic groups. However, the administration of both *B. adolescentis* and *B. bifidum* significantly enhanced the expression of ATG7 and LAMP2 while reducing PLIN2 levels. Based on existing evidence, we can conclude that ATG7 and LAMP2 expression may be inadequate under conditions of HFD feeding and STZ injection. The increased levels of ATG7 and LAMP2 associated with these probiotics may contribute to their protective effects against liver injury, as well as improvements in lipid profiles and liver function tests. Furthermore, these probiotics may promote lipolysis and lipophagy by diminishing PLIN2 expression, thereby exposing LDs for degradation through cellular mechanisms.

DGAT2 is an important enzyme involved in the synthesis of TG, and the buildup of TG in adipose tissue is a key factor in the development of obesity (Ning et al. 2017). Research conducted on obese mouse models has demonstrated that lowering DGAT2 expression through antisense oligonucleotide treatment improves hepatic steatosis, but simultaneously exacerbates the development of liver fibrosis (Schulze and McNiven 2023; 2019). Levin et al. (2007) discovered that fat deposition mediated by DGAT2 in glycolytic muscle specifically heightens insulin resistance in this tissue and may contribute to the progression of diabetes. Kantartzis et al. (2009) demonstrated that DGAT2 plays a key role in separating fatty liver from insulin resistance in humans. This discovery could be significant for preventing and treating insulin resistance and T2D in individuals with fatty liver. Furthermore, Rong et al. (2024) found that the inhibition of DGAT2 redirects diacylglycerol toward phospholipid synthesis, elevating the levels of phosphatidylethanolamine in the ER. This process results in decreased SREBP‐1 cleavage and a reduction in hepatic steatosis. Sterol‐regulatory proteins (SREBPs) encompass a group of transcription factors that are regulated and promote lipid synthesis in the liver (Shimomura et al. 1999). Research involving both cell cultures and animal models has demonstrated that a deficiency in SREBP1c lowers the risk of metabolic disorders, including atherosclerosis, obesity, and MAFLD. Sozen et al. (2021) demonstrated that a lack of SREBP1c influences the breakdown of LDs through autophagy in the context of oleic acid‐induced steatosis. Nguyen et al. (2021) discovered that SREBP‐1c impairs autophagic flux through the sulfhydration of ULK1, contributing to hepatic steatosis in mice fed an HFD. Their findings indicate a dual mechanism by which SREBP‐1c facilitates the accumulation of liver fat, characterized by both the activation of lipid biosynthesis and the inhibition of lipid degradation. Furthermore, elevated levels of nuclear SREBP‐1c were associated with an increased rate of hepatic fatty acid synthesis, ultimately resulting in steatosis in diabetic rats.

Here, we observed significantly elevated levels of FAS, DGAT2, and SREBP1 in the livers of rats fed an HFD and injected with STZ. In contrast, a substantial reduction in the expression of these genes was noted in rats that received treatments with either *B. adolescentis* or *B. bifidum*, as well as in those receiving a combination of these probiotics. The downregulation of lipogenic markers such as FAS, DGAT2, and SREBP1 suggests a potential reduction in de novo lipogenesis, further indicating improved lipid profiles. These findings imply that *B. adolescentis* and *B. bifidum*, along with their combined application, may protect against liver damage induced by STZ and HFD exposure by suppressing de novo lipogenesis. It is well established that assessing LC3 and p62/SQSTM1 levels is critical for interpreting autophagic activity (Siri et al. 2025; Mizushima 2007). A reduction in LC3 levels accompanied by an increase in p62 is indicative of suppressed autophagy, as observed in the HFD+STZ group. In contrast, an increase in LC3 levels together with a decrease in p62 reflects enhanced autophagic flux and efficient degradation of cargo within autolysosomes. This latter pattern was observed in both the monotherapy and combined therapy groups treated with *B. adolescentis* and *B. bifidum*.

Stereological analyses have shown that these probiotics mitigated diabetes‐ and obesity‐related pathological changes in the liver, including alterations in hepatic architecture and the proliferation of KCs. These findings might be indicative of the regenerative potential of *Bifidobacterium* strains, supporting their role in maintaining liver health and function. The complexity of metabolic regulation in obesity and diabetes is underscored by the selective influence of these probiotics on specific metabolic pathways, even when blood glucose levels do not significantly change. This highlights the intricate interplay between gut microbiota and metabolic processes, necessitating further investigation into the precise biochemical pathways involved.

## 5 Limitations and Future Directions

The present study has several limitations that should be acknowledged. First, gut microbiota composition was not analyzed; therefore, the specific microbial shifts induced by *B. adolescentis* and *B. bifidum* remain unknown. Second, although the sample size was calculated based on prior studies and power analysis, it was relatively modest, which may have limited the detection of subtle effects. Third, the study focused on short‐term interventions, and thus the long‐term safety and efficacy of these probiotics in MAFLD have yet to be determined. Finally, while LC3 (*Map1lc3b*) and p62 (*Sqstm1*) mRNA expression levels were assessed using qPCR, protein‐level analyses such as LC3‐II immunoblotting and p62 degradation assays were not performed. Due to financial constraints, we were unable to include these assays in the present work; however, future studies should incorporate them to directly evaluate autophagic flux and strengthen mechanistic conclusions.

Future studies should combine molecular, proteomic, and histological markers of autophagy to better characterize the mechanistic links between probiotics and hepatic lipid metabolism. Integrating gut microbiota profiling and metabolomic analysis would also clarify the contributions of the gut–liver axis to the observed effects. In addition, testing these interventions in other MAFLD models, including those with varying metabolic backgrounds and longer follow‐up periods, would help determine their broader applicability and potential translational value. Future research should focus on elucidating these mechanisms and exploring the applicability of *Bifidobacterium* strains as dietary interventions for individuals with metabolic disorders. Understanding these pathways could lead to targeted probiotic therapies that enhance metabolic health and prevent the progression of obesity and T2D through dietary means.

## 6 Conclusion

In conclusion, our findings provide compelling evidence for the beneficial effects of *B. adolescentis* and *B. bifidum* in modulating lipid metabolism and preventing liver damage in the setting of obesity and diabetes. The observed changes in the expression of key metabolic regulators highlight the importance of these probiotics in potentially facilitating autophagic processes and restoring metabolic homeostasis. Future studies are warranted to elucidate the precise pathways involved and to explore the clinical applications of these probiotics as dietary interventions in metabolic disorders.

## Author Contributions

**Mahtab Mehboodi:** writing – original draft preparation. **Mohammad Mehdi Alinaghi:** writing – original draft preparation. **Maryam Behmanesh:** investigation. **Mohammad Hosein Kolahkaj:** investigation. **Milad Pour Mohammad Ali Namdari:** writing – review and editing. **Hadis Khanbabaie:** writing – original draft preparation. **Arezoo Harsjian:** investigation. **Narges Namavar:** validation, formal analysis. **Saman Rabiei:** formal analysis. **Maryam Sadat Pishva:** methodology. **Payam Baziyar:** conceptualization**. Sara Rashki Ghaleno:** project administration. **Mohammad Hasan Maleki:** supervision, writing – review and editing.

## Ethics Statement

The study was approved by the Institutional Animal Ethics Committee of Shiraz University of Medical Sciences, Shiraz, Iran study (Ethics Code IR.SUMS.AEC.1403.046).

## Conflicts of Interest

The authors declare no conflicts of interest.

## Data Availability

All data sets analyzed during the current study are available from the corresponding author on reasonable request.
